# Supplementary material for: Microbial species and intraspecies units exist and are maintained by ecological cohesiveness coupled to high homologous recombination
Source: Nat Commun. 2024 Nov 15;15:9906. doi: 10.1038/s41467-024-53787-0 (PMC11568254; doi:10.1038/s41467-024-53787-0)
Supplement: Supplementary file 1 — Supplementary Information [file 41467_2024_53787_MOESM1_ESM.pdf]

SUPPLEMENTARY FIGURES

TITLE: Microbial species and intraspecies units exist and are maintained by ecological cohesiveness coupled to high homologous recombination.

AUTHORS: Roth E. Conrad[1,*], Catherine E. Brink[1,*], Tomeu Viver[2,3,*], Luis M. Rodriguez-R[4], Borja Aldeguer-Riquelme[1], Janet K. Hatt[1], Stephanus N. Venter[5], Ramon Rossello-Mora[2], Rudolf Amann[3], and Konstantinos T. Konstantinidis[1]

[1]Georgia Institute of Technology, Atlanta, GA, USA.

[2]Mediterranean Institutes for Advanced Studies (IMEDEA, CSIC-UIB), Esporles, Spain

[3]Max Planck Institute for Marine Microbiology, Bremen, Germany.

[4]University of Innsbruck, Innsbruck, Austria.

[5]University of Pretoria, Pretoria, South Africa.

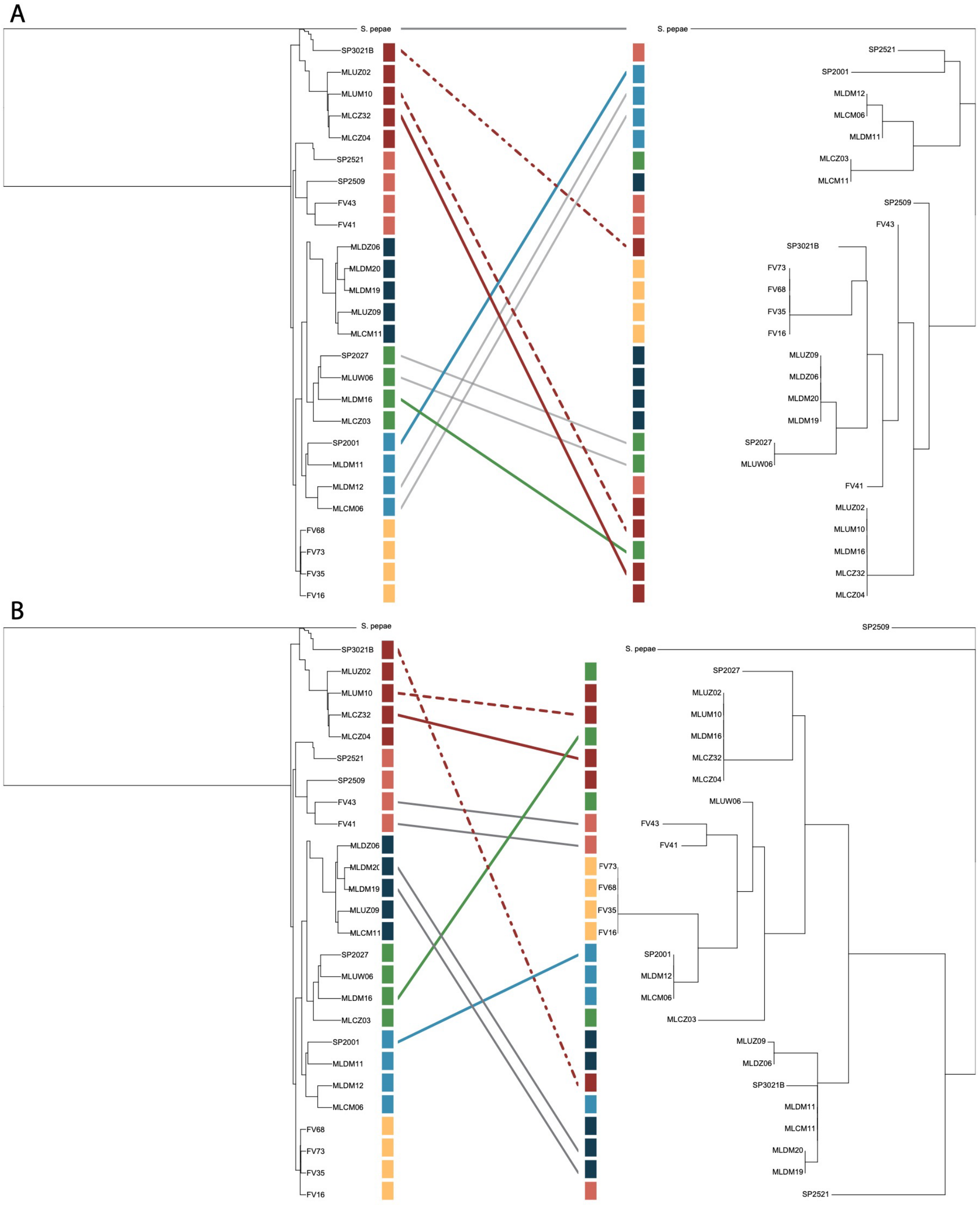

Figure S1: Phylogenetic trees showing examples of recent recombination of functional genes between *Sal. ruber* phylogroups. Two tanglegrams are used to illustrate the recombination in the regions identified in Figure 2A as "recombination outside genomovar" by the blue arrows. The pruned ANI tree (left, Panels A and B) is compared to two gene trees: one from a region at 782 574 bp (right, Panel A; an adenine deaminase; closest match: UniRef100_Q2S006) and the second from the region identified at 1 261 301 bp (right, Panel B; an ATPase beta subunit or *atpD*; closest match: UniRef100_Q2RZV3) along the reference genome (MLCZ32). The genes have undergone recent gene exchange between distinct ANI genomovars (of different phylogroups) as reflected by the clustering, at high nucleotide identity, with different genomovars in the gene relative to the ANI tree (a few examples are denoted by the colored lines). A single gene was selected from each region to generate the alignment and gene tree using MEGA11 [1]. The base tanglegrams were constructed in R language. Phylogroups are colored as in the ANI tree.

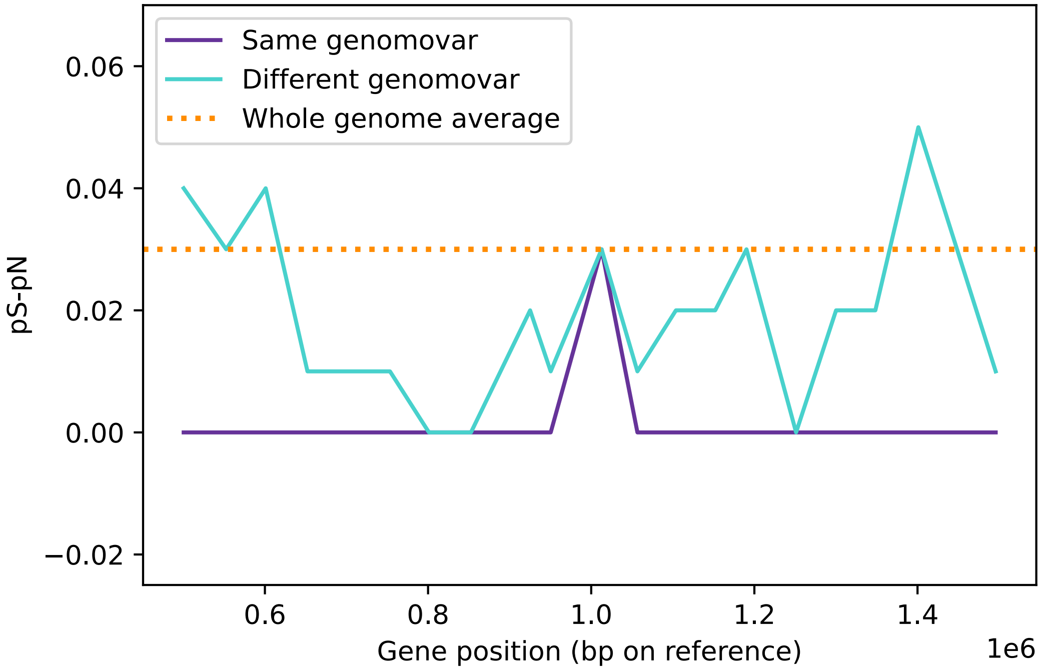

Figure S2: Evidence for the lack of positive (adaptive) selection in areas of recent recombination. The plot shows the ratio between the proportion of non-synonymous and synonymous substitutions along the genomic region shown in Figure 2A. The ratio fluctuates between different genomovars, but is never greater than 0.4, which indicates that it is unlikely that any of the corresponding genes are experiencing adaptive evolution. The pN/pS ratio was calculated using the script developed by T. Zhu, 2020 available at https://github.com/zhutao1009/dnds.git. The whole genome average was calculated using 13 genes spread across the whole genome at intervals of ~250000 bp and are supplemented by ratios calculated for the same and different genomovar for 20 genes at ~50000 bp across the segment shown in Figure 2A.

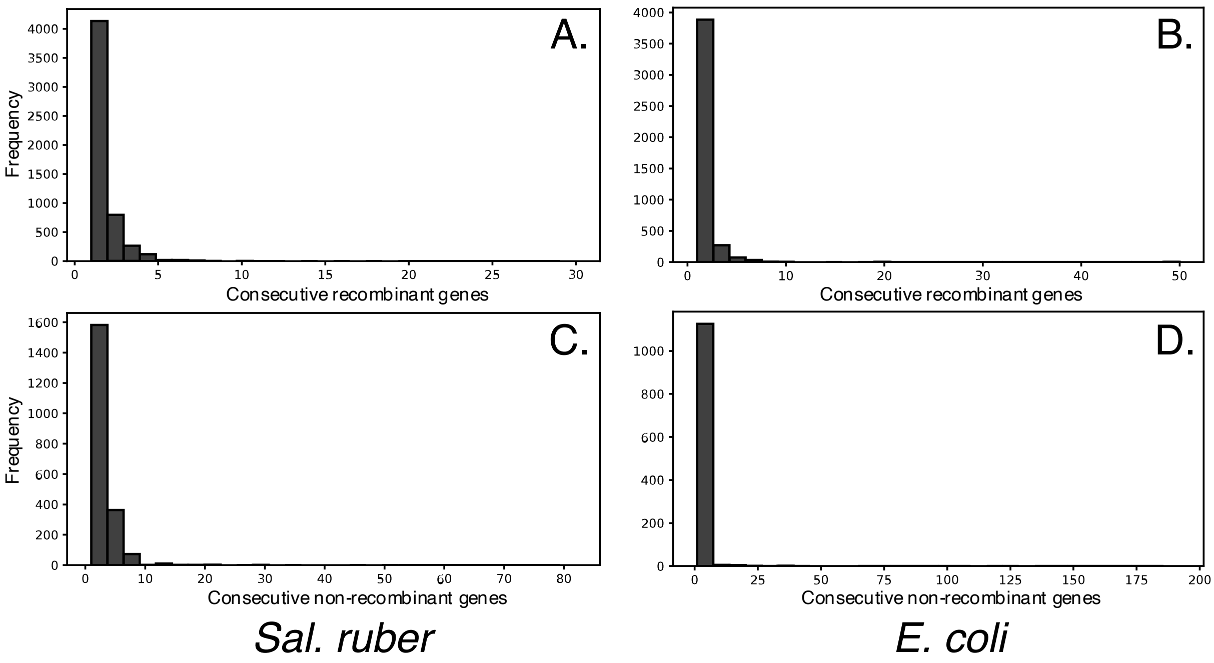

Figure S3: Length distribution of recombinant fragments between the genomes. The graphs show the frequency and distribution of consecutive recombinant genes as a proxy for the length distribution of recombinant fragments (A and B) and consecutive non-recombinant genes (C and D) for the *Sal. ruber* (A and C) and *E. coli* (B and D) genomes used in this study.

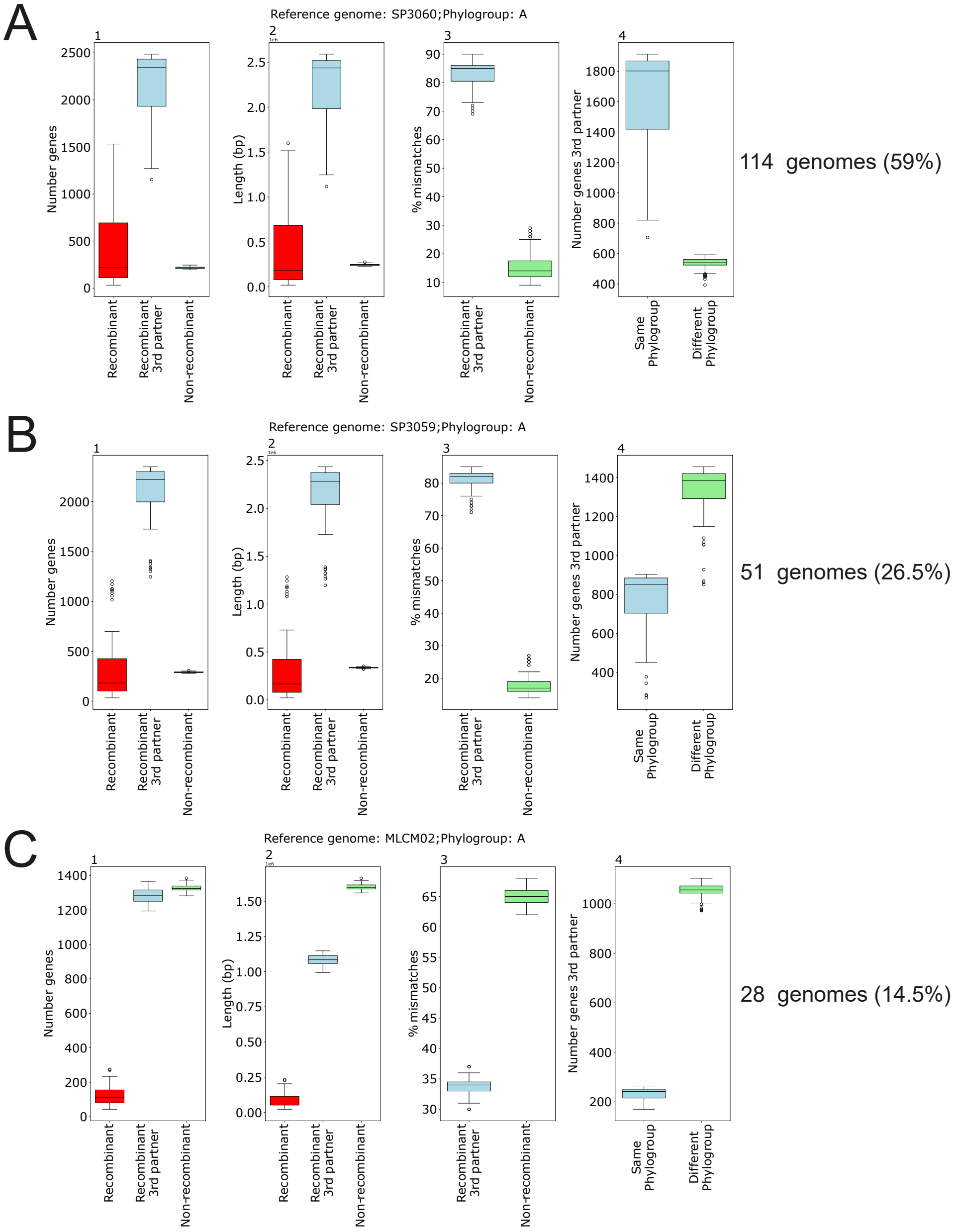

Figure S4: Assessing the relative impact of recombination as a cohesive vs diversifying force. Each set of graphs shows the results for a comparison of a reference genome of one genomovar against all genomes of different genomovars and consists of 4 plots that show the number of genes (1), their length (2), the percentage of mismatches between each genome pair (3), and the most likely recombining genome (based on best match) for the genes that recombine with a 3rd partner (4). For each plot, the genes were grouped into three categories: recombinant genes (red, proxy for cohesion force), recombinant genes with a 3rd partner (blue, proxy for diversification force) and non-recombinant genes (green, proxy for mutations). Three different main patterns were identified across the total of 193 *Sal. ruber* genomes evaluated, and one example of each of them is shown in the figure. Panel A: a reference genome that showed a higher strength of recombination as a force of cohesion at the phylogroup level. Panel B: a reference genome that showed higher strength of recombination as a force of diversification at the phylogroup level (but cohesive at the species level). Panel C: a reference genome that showed mostly non-recombined genes. The numbers at the right side of each row indicate the number of genomes that showed the pattern. See text for further details.

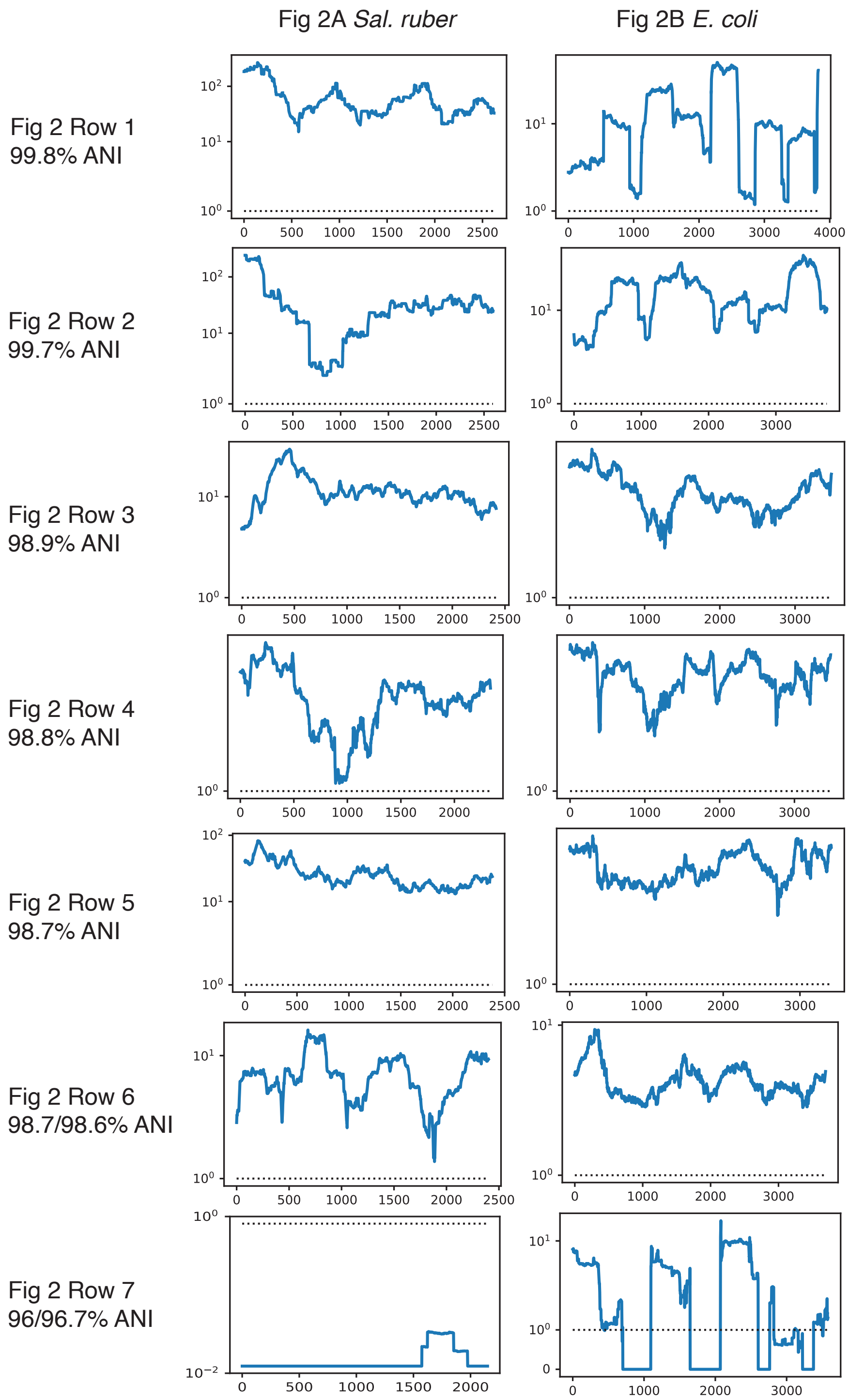

Figure S5. Recombination affects every segment of the genome and is higher than mutation. The same reference and query genomes, with the same order, as shown in Figure 2 were used. The graphs show the recombination to mutation ratio (r/m, y-axes) across the reference genome in pairwise genome comparisons for each shared gene across the genome (x-axes). The ratio was estimated using our empirical approach described in the main text. Note that the ratio is often larger than 1, and even if there are segments of the genome with ratio <1 in some genome comparisons, other genomes show ratios >1 for the same segments, revealing that recombination is genome-wide (not spatially biased) at the whole-population/species level.

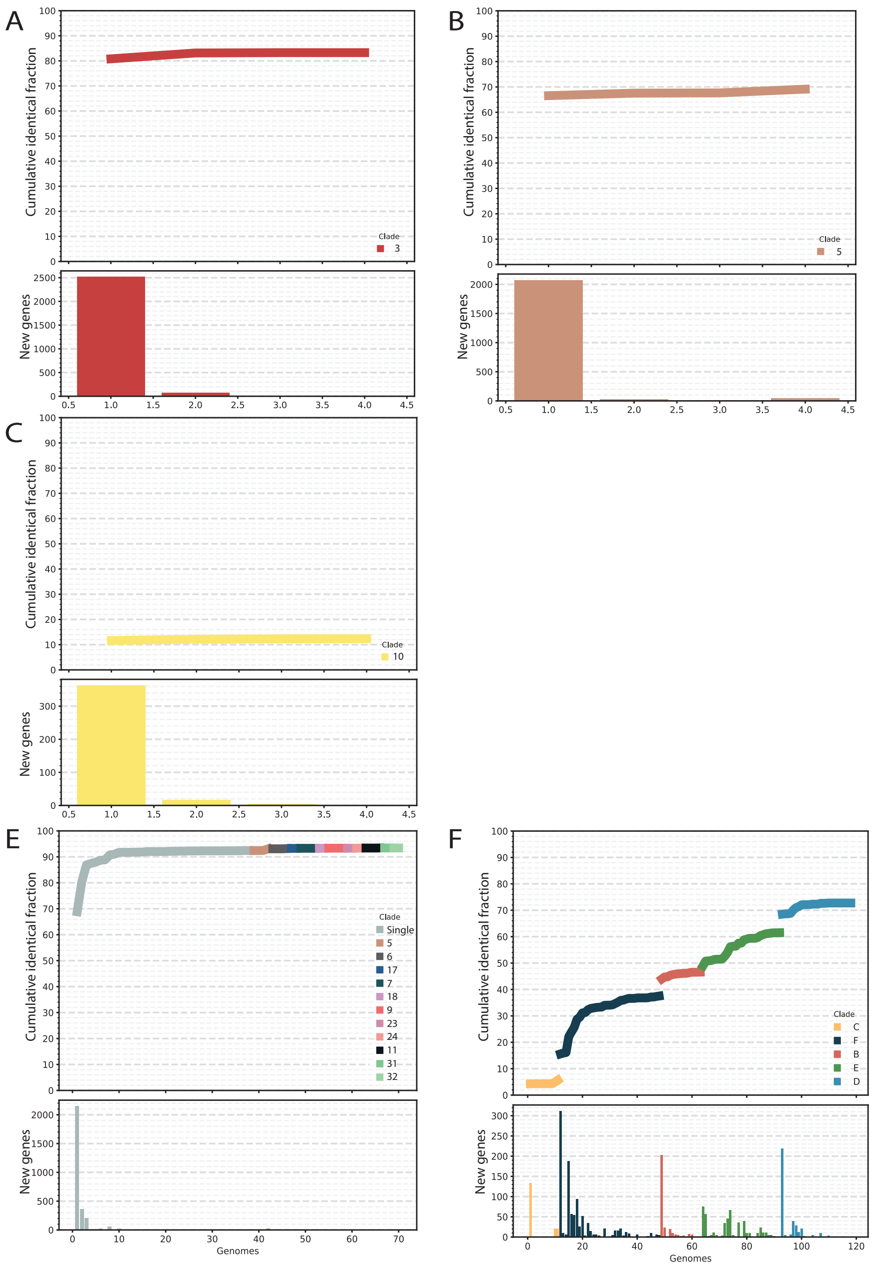

Figure S6. Examples of cumulative identical gene fraction curves for one vs. many genome comparisons. The y-axis shows the fraction of identical genes among all genes in the reference genome (top panels) and the number of new identical genes (bottom panels) for each genome added (x-axis), i.e. number of identical genes as a function of the number of genome partners included in the analysis. The same *Sal. ruber* reference genome used in Figure 5A (Phylogroup A, genomovar 3) is compared against the groupings used in Figure 5 which were (A) genomes within the same genomovar (genomovar 3), (B) genomes from a different genomovar (genomovar 5) within the same phylogroup (phylogroup A), (C) genomes in a different genomovar (genomovar 10) from a different phylogroup, (E) genomes within the same phylogroup (phylogroup A) excluding genomes from the same genomovar (genomovar 3), and (F) genomes within the same species excluding genomes from the same phylogroup (phylogroup A). An example corresponding to panel D (i.e., one genome against genomes of a different species) is not shown because only a single *Sal. pepae* genome was available. Figure 5 essentially shows the final (far right) point of the curve for each one vs. many genome comparison and the abovementioned groups (A though E).

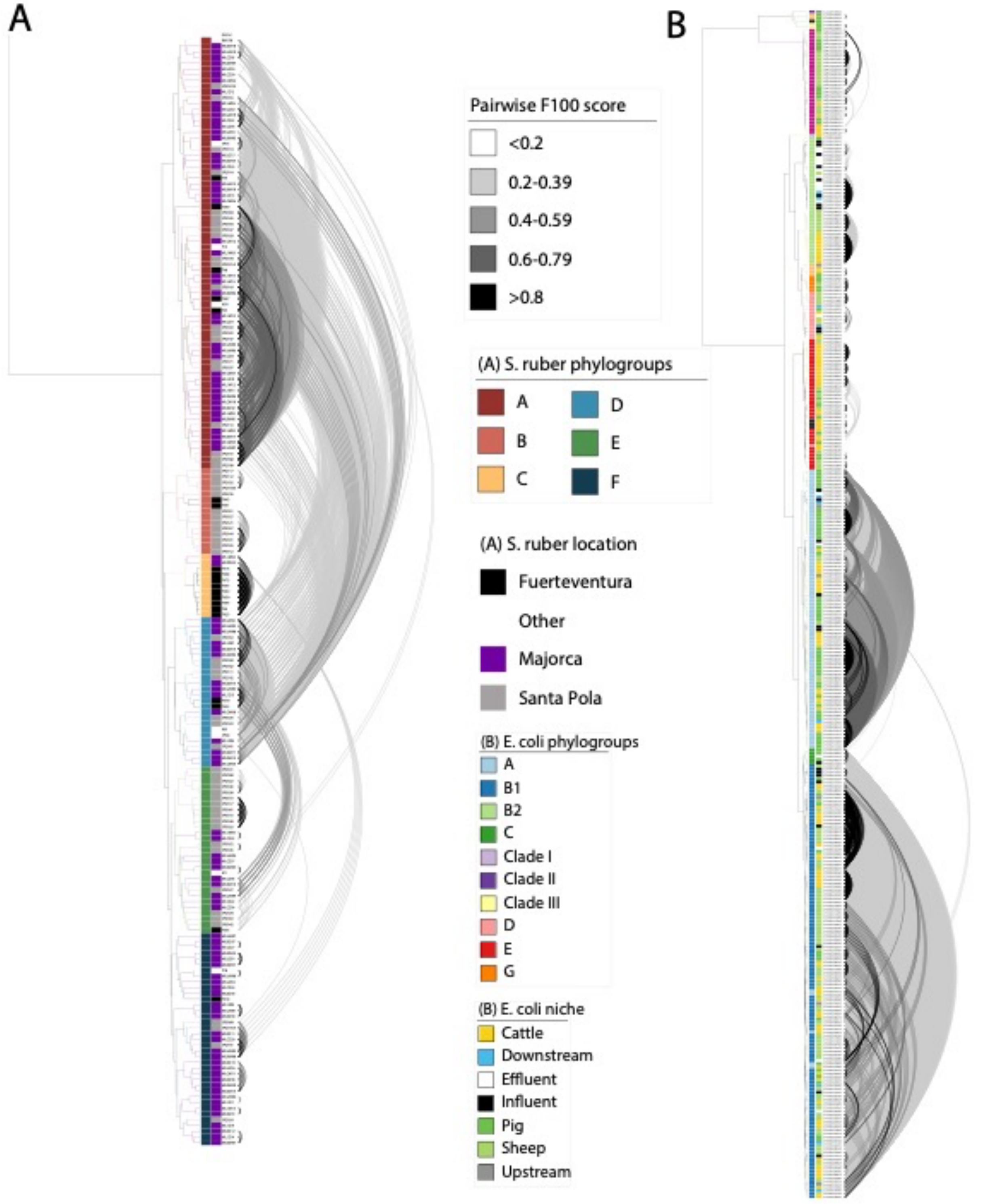

Figure S7. ANI trees showing the phylogroup (clade) structure for each species studied and recent gene exchange. The frequency of 100% identical RBM genes to total RBM genes found (F100), a proxy for recent recombination, for each genome pair is shown by connecting lines shaded along a grayscale gradient for *Sal. ruber* (A) and *E. coli* (B). Genome pairs with F100 value close to zero (or zero) are not

shown. For *Sal. ruber*, if the name of a genome starts with ML, FV or SP, it denotes that the corresponding isolate originated from the Mallorca, Canary Islands or Santa Pola (mainland Spain) sites. Site (for *Sal. ruber*) and niche of isolation (for *E. coli*) information are also shown (see Figure key). Note that these two factors do not appear to have an major effect on phylogroup clustering.

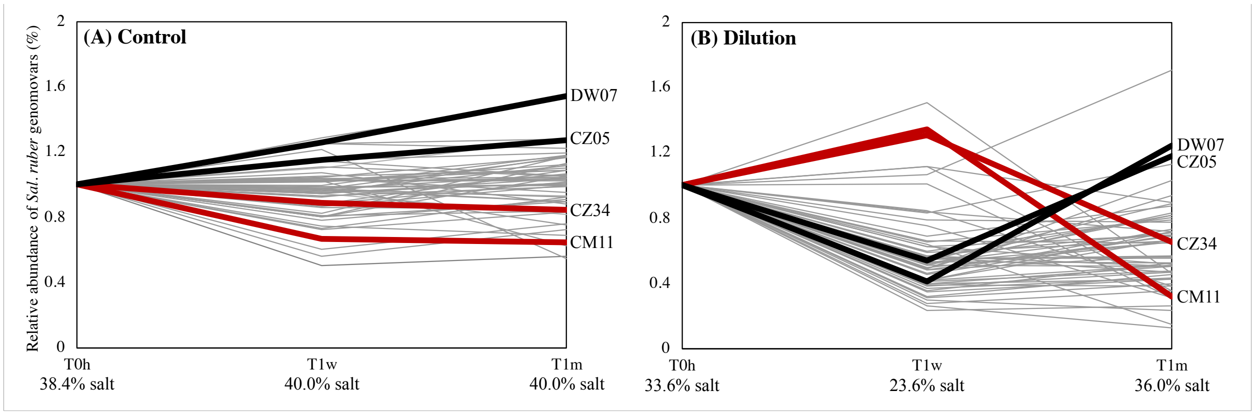

Figure S8. *Sal. ruber* genomovar abundance dynamics over the one-month period of experimental manipulation of salinity. Each line represents a (distinct) genomovar and shows its relative abundance as a fraction of the total *Sal. ruber* population, based on the number of metagenomic reads uniquely recruited by the representative genome of the genomovar (y-axes), against the three metagenomic sampling time points (x-axes) for each of the two separate experimental ponds used (panel title on top). For the dilution pond, the salt concentration was reduced from 33.6 to 12.0% by the addition of freshwater at time zero (0 h); the control pond experienced no treatment and was at salt-saturation conditions during sampling. Note that the two genomovars in red strongly prefer low salt conditions while the two genomovars in black grew better at salt-saturated conditions, while all four genomovars showed much less change in abundance in the control pond. Figure is modified from [2].

Supplementary References

1 Tamura, K., Stecher, G. & Kumar, S. MEGA11: Molecular Evolutionary Genetics Analysis Version 11. *Mol Biol Evol* **38**, 3022-3027 (2021). https://doi.org/10.1093/molbev/msab120

2 Viver, T. *et al.* Towards estimating the number of strains that make up a natural bacterial population. *Nat Commun* **15**, 544 (2024). https://doi.org/10.1038/s41467-023-44622-z
