# Supplementary material for: Microbial species and intraspecies units exist and are maintained by ecological cohesiveness coupled to high homologous recombination
Source: Nat Commun. 2024 Nov 15;15:9906. doi: 10.1038/s41467-024-53787-0 (PMC11568254; doi:10.1038/s41467-024-53787-0)
Supplement: Supplementary file 2 — Description of Additional Supplementary Files [file 41467_2024_53787_MOESM2_ESM.pdf]

Description of Additional Supplementary Files:

Supplementary Data 1: Supplementary table that has the information of all genomes used in the study.
